# Isolation and Characterization of Multipotent Canine Urine-Derived Stem Cells

**DOI:** 10.1155/2020/8894449

**Published:** 2020-09-30

**Authors:** Yan Xu, Tao Zhang, Yang Chen, Qiang Shi, Muzhi Li, Tian Qin, Jianzhong Hu, Hongbin Lu, Jun Liu, Can Chen

**Affiliations:** ^1^Key Laboratory of Organ Injury, Aging and Regenerative Medicine of Hunan Province, Changsha, China 410008; ^2^Hunan Engineering Research Center of Sports and Health, Changsha, China 410008; ^3^Xiangya Hospital-International Chinese Musculoskeletal Research Society Sports Medicine Research Centre, Changsha, China 410008; ^4^Department of Sports Medicine, Xiangya Hospital, Central South University, Changsha, China 410008; ^5^Department of Spine Surgery, Xiangya Hospital, Central South University, Changsha, Hunan, China 410008; ^6^Department of Limbs (Foot and Hand) Microsurgery, Affiliated Chenzhou No.1 People's Hospital, Southern Medical University, Chenzhou, China 423000; ^7^Department of Orthopedics, Xiangya Hospital, Central South University, Changsha, China 410008

## Abstract

Current cell-based therapies on musculoskeletal tissue regeneration were mostly determined in rodent models. However, a direct translation of those promising cell-based therapies to humans exists a significant hurdle. For solving this problem, canine has been developed as a new large animal model to bridge the gap from rodents to humans. In this study, we reported the isolation and characterization of urine-derived stem cells (USCs) from mature healthy beagle dogs. The isolated cells showed fibroblast-like morphology and had good clonogenicity and proliferation. Meanwhile, these cells positively expressed multiple markers of MSCs (CD29, CD44, CD90, and CD73), but negatively expressed for hematopoietic antigens (CD11b, CD34, and CD45). Additionally, after induction culturing, the isolated cells can be differentiated into osteogenic, adipogenic, chondrogenic, and tenogenic lineages. The successful isolation and verification of USCs from canine were useful for studying cell-based therapies and developing new treatments for musculoskeletal injuries using the preclinical canine model.

## 1. Introduction

Stem cells and tissue-derived stromal cells stimulate the repair of degenerated and injured tissues, motivating a growing number of cell-based therapies in the musculoskeletal field [1, 2]. Mesenchymal stem cells (MSCs) showed a good self-renew ability and were capable of differentiating into the progeny of several lineages, including osteoblasts, chondrocytes, adipocytes, fibroblasts, tenocytes, and myoblasts [3–7]. Thus, it was the most commonly used cell source in cell-based therapies. In recent years, preclinical and clinical studies have determined that the MSCs isolated from the bone marrow, peripheral blood, adipose tissue, synovium, and periosteum [3, 8, 9] have the therapeutic potential for the regeneration of injured musculoskeletal tissues, such as the bone, cartilage, tendon, enthesis, and intervertebral disc [10–14]. However, these types of MSCs are restricted by the invasive and painful harvesting procedures, which may cause donor site morbidity and limit their use for autogenous approaches. Therefore, finding a stem cell harvested without invasive and painful procedures would help us escape from the dilemma of the current cell-based therapies.

Urine-derived stem cells (USCs) isolated from urine have received significant attention and been studied as a promising candidate for the development of new cell-based therapies owning to their multilineage potential (differentiation into osteocytes, chondrocytes, adipocytes, neurocyte, myocytes, and endothelial cells) and sufficient proliferation capacities [15. 16]. The advantages of USCs include noninvasive and low-expense harvesting as well as being considered ethical. Additionally, USCs have been isolated from autologous urine which do not induce immune responses or rejection [15, 16]. Therefore, USCs are considered as an attractive alternative source for cell-based therapies to enhance musculoskeletal tissue regeneration.

Currently, most of the cell-based therapies on musculoskeletal tissue regeneration were determined in rodent models. A direct translation of those promising cell-based therapies to humans exists a significant hurdle. For solving this problem, a number of large animal species have been used by researchers to bridge the gap from rodents to humans [17–21]. Among these large animal species, canine represents a compelling model for translational studies. Compared with the rodents, dogs are large, long-lived, genetically diverse, and share many physiological and biochemical similarities with humans. Until now, canine models have been successfully used to develop adult bone marrow transplantation, gene therapy, and allograft rejection protocols for use in humans [22–24]. In addition, dogs have good compliance and response to learned behaviors, such as treadmill exercise; it was used to evaluate new therapies for cardiovascular and orthopedic diseases [25, 26]. Based on these benefits, the preclinical canine models in osteoarthritis, anterior cruciate ligament reconstruction, rotator cuff repair, meniscal injury, and nonunion fracture have been developed and described in recent articles [27–33], which can be used to bridge the gap from rodents to humans. Although USCs have been successfully isolated in humans, no report has described the protocol of isolating USCs from canine (cUSCs). Therefore, it is imperative to isolate cUSCs to facilitate future studies on regenerative strategies for musculoskeletal tissue injuries.

In this study, we described the isolation and identification of cUSCs for the first time, and its morphology at different passages, surface markers, proliferation capacity, clonogenicity, and multilineage differentiation potential were investigated in vitro. The protocol developed for isolating cUSCs is an essential step to extrapolate USC-based therapy from a preclinical canine model for clinical management of tissue injuries.

## 2. Materials and Methods

### 2.1. Ethics Statement

The local animal ethics committee approved the experimental protocol for the use of beagle dogs in this study.

### 2.2. Canine USC Isolation and Expansion

**Figure 1:**
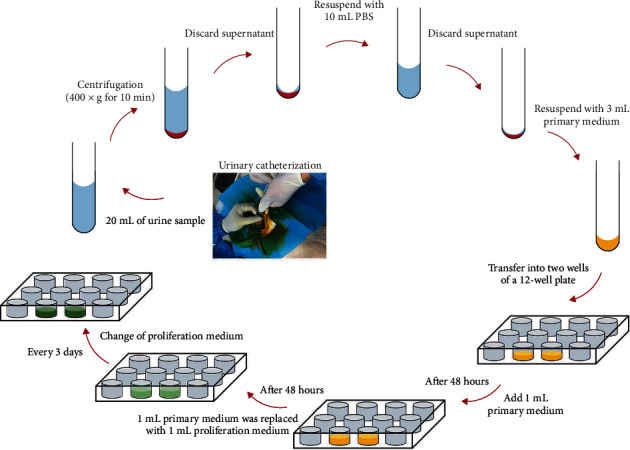
Schematic diagram showing the procedure of canine USC isolation and culture.

The procedures of cUSC isolation and culture are depicted in Figure 1. Briefly, three healthy dogs were anesthetized with 3% pentobarbital sodium (0.15 mL/kg), and about 20 mL of urine sample was obtained from each dog with a sterile catheter. After centrifugation at 400 × g for 10 min, the supernatant was discarded, and about 1 mL of the remaining liquid in the tube was gently resuspended with 10 mL PBS containing 1% antibiotic-antimycotic (Gibco, USA). And then, the mixture was centrifuged at 200 × g for 10 min, and the supernatant was discarded. Three milliliters of primary medium (Table 1) was added into the remaining liquid, and the cell pellet was gently resuspended. The cell supernatant was averagely transferred into two wells of a 12-well plate. After the first 48 h, each well was added with 1 mL of primary medium. At the second 48 h, 1 mL of primary medium was replaced with 1 mL of fresh proliferation medium (Table 2). The whole medium was changed with a proliferation medium every 3 days. The cells were passaged when reaching 80-90% confluence. Passage 3 cells were used for further experiments.

### 2.3. Colony-Forming Unit (CFU) Assay

The CFU-F assay was performed to evaluate the clonogenicity of the isolated canine-derived cells. Briefly, canine USCs were plated on a 6-well plate in triplicate at 20 cells per well. After 10 days of cultivation, the cells were stained with 1% crystal violet (Solarbio, CHN), and the stained colonies bigger than 2 mm were counted. The CFU-F assay was performed independently in three dogs.

### 2.4. Cell Proliferation

The proliferation of isolated cells was evaluated at time points of 1, 3, 5, 7, and 9 days using the Cell Counting Kit-8 (CCK-8) (7seabiotech, China). Briefly, canine USCs were plated in a 96-well plate at 2 × 10^3^ cells per well and incubated at 37°C with 5% CO_2_. At the desired time point, the cells in each well were incubated with 10 *μ*L of CCK-8 reagent and 100 *μ*L serum-free medium. After incubating the plate at 37°C with 5% CO_2_ for 2 h, the absorbance at 450 nm was recorded using a microplate reader (Varioskan LUX, Thermo, USA). Each experiment was performed in four replicates. The cell proliferation assay was performed independently in the three dogs.

### 2.5. Surface Markers of Isolated Cells

The surface markers of isolated cells were analyzed by flow cytometry analysis. Briefly, the isolated cells (1 × 10^6^ cells, passage 3) from the three dogs were, respectively, suspended in 100 *μ*L phosphate-buffered saline (PBS) containing 10 *μ*g/mL antibodies for PE-conjugated CD29 (303003, BioLegend, USA), PE-conjugated CD44 (103024, BioLegend, USA), PE-conjugated CD90 (561970, BD Biosciences, USA), PE-conjugated CD105 (bs-0579R-PE, Bioss, CHN), FITC-conjugated CD73 (bs-23233R-FITC, Bioss, CHN), PE-conjugated CD34 (559369, BD Biosciences, USA), and FITC-conjugated CD45 (11-5450-42, eBioscience, USA). After incubation for 30 min at 4°C, the cells were washed with PBS and then resuspended in 500 *μ*L of PBS for analysis. As for CD11b, the isolated cells (1 × 10^6^ cells, passage 3) were suspended in 100 *μ*L phosphate-buffered saline (PBS) containing 10 *μ*g/mL CD11b (MA5-16604, eBioscience, USA). After incubation for 30 min at 4°C, the cells were washed with PBS and then labeled with goat antirabbit IgG (H + L) cross-adsorbed secondary antibody, FITC (F-2765, Invitrogen), at a dilution of 1 : 500 for 1 h at room temperature. Cell fluorescence was evaluated by flow cytometry using a DxP Athena™ flow cytometry system (Cytek) and analyzed with FlowJo 10 software (Tree Star, USA).

### 2.6. Osteogenic Differentiation

The isolated cells were cultured in complete medium at 5000 cells/cm^2^ in 6-well plates. When the cultured cells reached 80%-90% confluence, three wells of cells were cultured with basal complete medium (as the control group), and the other three wells of cells were cultured with osteogenic differentiation medium (MUBMD-90021, Cyagen, CHN) (as the osteogenic group). The medium was changed every 3 days. After 7-day culture, quantitative real-time polymerase chain reaction analysis (qRT-PCR) was performed for evaluating the expression of osteogenic genes (Runx2, Spp1, Bglap) in the cells. The primer sequences were listed in Table 3. Meanwhile, Runx2 expression was evaluated by immunofluorescence assay using the anti-Runx2 antibody (ab76956, Abcam, USA). Additionally, Alizarin Red was used to stain the calcium nodules for assessing osteogenic differentiation of isolated cells after a 21-day culture. To conduct Alizarin Red assay, the cells were washed twice with PBS, followed by 10 min fixation in 70% ethanol and incubated in 0.5% Alizarin Red solution for 30 min, followed by PBS washing to remove the residual dye. The images of stained cells were obtained using a fluorescence microscope and a light microscope, respectively.

### 2.7. Adipogenic Differentiation

The isolated cells were cultured in complete medium at 5000 cells/cm^2^ within 6-well plates. When the cultured cells reached 95-100% confluence, three wells of cells were cultured with basal complete medium (as the control group), and the other three wells of cells were cultured with adipogenic differentiation medium (MUBMD-90031, Cyagen, CHN) (as the adipogenic group). The medium was changed every 3 days. After 7-day culture, qRT-PCR was performed for evaluating the expression of adipogenic genes (PPAR*γ*, FABP4, LPL) in the cells. Meanwhile, the PPAR*γ* expression was evaluated by immunofluorescence assay using an anti-PPAR*γ* antibody (ab45036, Abcam, USA). The primer sequences were also listed in Table 3. Additionally, Oil red O was used to stain the neutral lipid vacuoles within cells for assessing the adipogenic differentiation of isolated cells after a 21-day culture. The steps for Oil red O staining assay are described below: the cells were washed twice with PBS and then fixed with 70% ethanol for 20 s. After that, they were incubated with filtered 0.3% Oil red O solution for 15 min, followed by thorough washing with PBS. The images of stained cells were viewed using a light microscope.

### 2.8. Chondrogenic Differentiation

The isolated cells were cultured in complete medium at 5000 cells/cm^2^ within 6-well plates. When the cultured cells reached 80%-90% confluence, three wells of cells were cultured with a basal complete medium (as the control group), and the other three wells of cells were cultured with a chondrogenic differentiation medium (MUBMD-90041, Cyagen, CHN) (as the chondrogenic group). The medium was changed every 3 days. After 7-day culture, qRT-PCR was performed for evaluating the expression of chondrogenic genes (Sox9, Acan, Col2a1) in the cells. The primer sequences are listed in Table 3. Meanwhile, Sox9 expression was evaluated by immunofluorescence assay using the anti-Sox9 antibody (PA5-23383, Invitrogen, USA). Additionally, Alcian blue was used to stain the deposition of proteoglycan around the cells for assessing the chondrogenic differentiation of isolated cells after a 21-day culture. The Alcian blue staining assay was performed by the following steps: the cells were washed twice with PBS and then fixed with 70% ethanol for 20 s. After that, they were incubated with Alcian blue solution for 30 min, followed by thorough washing with PBS. The images of stained cells were obtained using a fluorescence microscope and a light microscope, respectively.

### 2.9. Tenogenic Differentiation

The isolated cells were cultured in complete medium at 5000 cells/cm^2^ within 6-well plates. When the cultured cells reached 80%-90% confluence, three wells of cells were cultured in basal medium alone (as the control group) or supplemented with 50 ng/ml BMP-12 (PeproTech) (as the tenogenic group). The medium was changed every 3 days. After 7-day culture, qRT-PCR was performed for evaluating the expression of tenogenic genes (Tnmd, Scx, and Mkx) in the cells. The primer sequences were listed in Table 3. Meanwhile, Tnmd expression was evaluated by immunofluorescence assay using the anti-Tnmd antibody (ab81328, Abcam, USA). The images of stained cells were obtained using a fluorescence microscope.

### 2.10. Statistical Analysis

All quantitative data are presented as mean ± standard deviation. The comparison of the two groups was done using a two-tailed, unpaired Student's *t*-test, and the comparison of multiple groups was done using a one-way factorial analysis of variance (ANOVA) followed by the comparison of individual means with Tukey's test. The statistical analyses were performed using the SPSS 25.0 software (SPSS, USA). *p* < 0.c05 was regarded as statistically significant. All the experiments have been performed at least three biologically independent replicates.

## 3. Results

### 3.1. Morphology and Proliferation of Urine-Derived Cells

**Figure 2:**
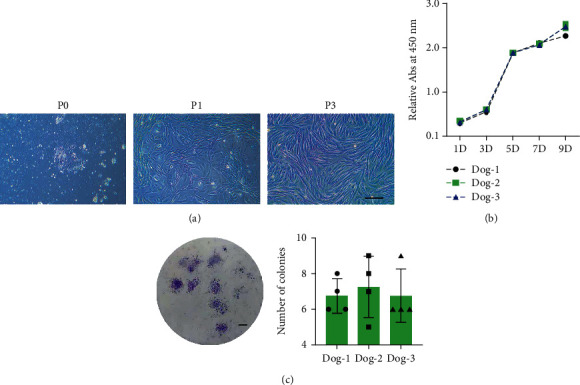
(a) Morphology of cells isolated from canine urine at different passages. At P0, spindle-shaped or round-shaped cells were observed. At P1, spindle-shape and fibroblast-like morphology cells were observed. Scale bars = 200 *μ*m. (b) The proliferation of urine-derived cells was determined by CCK-8 assay. Four samples were measured for each time point. The experiment was performed independently in the three dogs. (c) Colony-forming unit assay of the isolated cells after 10 days of culture. Scale bars = 2 mm.

At days 5–7 after primary culture, adherent cells and colonies with spindle-shape or round-shape were observed, and lots of dead sperm were suspended in the medium (Figure 2(a)). In passage 1, most of the isolated cells exhibited a spindle-shape and fibroblast-like morphology, and few of them still displayed round-shape (Figure 2(a)). At passage 2 or 3, the canine urine-derived cells exhibited a homogeneous spindle-shaped morphology (Figure 2(a)). CCK-8 assay indicated that passage 3 of the isolated cells proliferated with a high rate within the first 5 days (Figure 2(b)).

### 3.2. Clonogenicity of Urine-Derived Cells

The clonogenicity of the isolated urine-derived cells was determined using the CFU assay. After 10 days of culture, cells isolated from urine formed adherent cell colonies (Figure 2(c)). Statistically, the isolated cells formed 6.92 ± 1.31 colonies at a density of 40 cells per well (Figure 2(c)).

### 3.3. Surface Marker of Urine-Derived Cells

**Figure 3:**
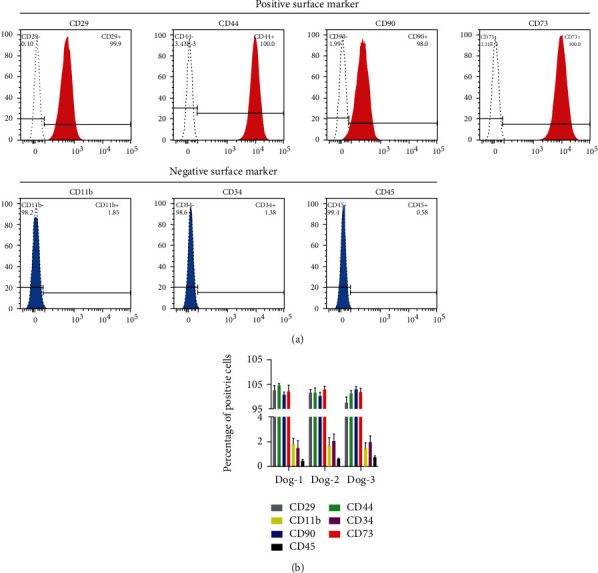
Flow cytometry for the isolated cells. (a) Representative histograms demonstrating positive and negative staining of urine-derived cells from a single dog. (b) Percentage of positive cells for the urine-derived cells isolated from three canine donors.

Flow cytometric analysis showed that passage 3 urine-derived cells positively expressed multiple markers of MSCs (CD29, CD44, CD90, and CD73), but negatively expressed for hematopoietic antigens (CD11b, CD34, and CD45) (Figure 3). These immunophenotypic profiles were in accordance with the criteria for defining MSCs proposed by the International Society for Cellular Therapy (ISCT) [34].

### 3.4. Osteogenic Differentiation Potential

**Figure 4:**
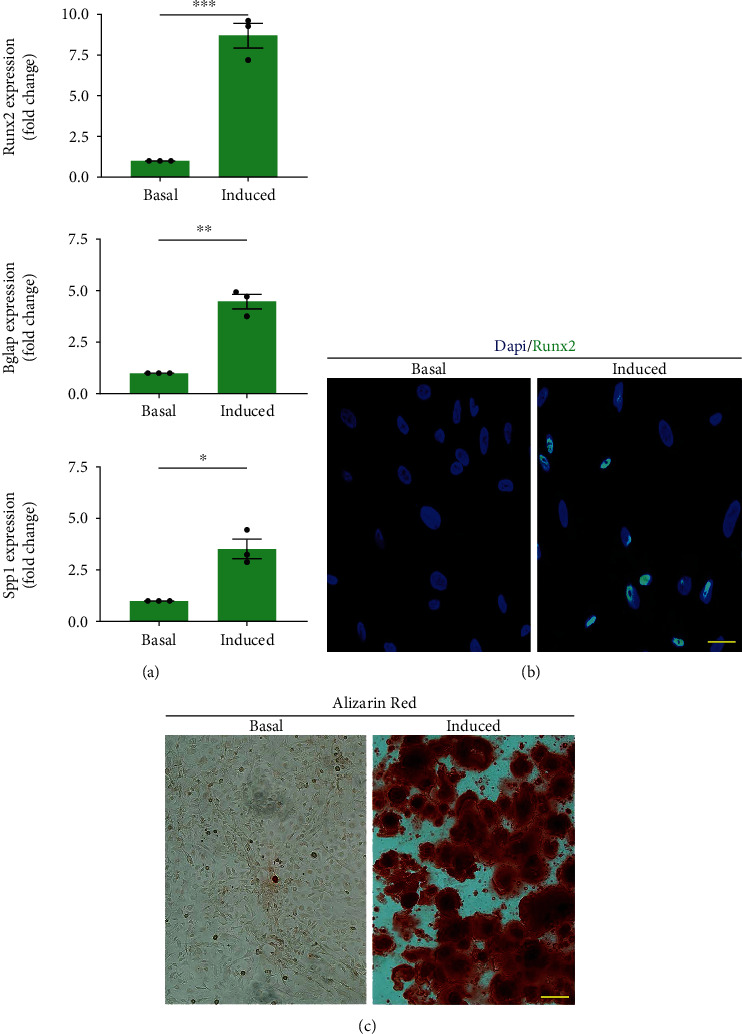
Osteogenic differentiation of urine-derived cells in vitro. (a) Osteogenic gene (Runx2, Bglap, SPP1) expression compared between osteogenic medium and its respective basal cultures. ^∗∗^*p* < 0.01, ^∗∗∗^*p* < 0.001. Runx2: Runt-related transcription factor 2; Bglap, bone *γ*-carboxyglutamate protein; Spp1, secreted phosphoprotein. (b) The Runx2 protein expression of the urine-derived cells after 7 days of osteogenic induction. Scale bars = 15 *μ*m. (c) Alizarin Red staining of cells after 21 days in osteogenic or basal medium. Calcium nodules were seen in the osteogenic induced medium. Scale bars = 20 *μ*m.

After 7 days of culture, the cells in osteogenic induced medium showed a significantly higher mRNA expression level of Runx2, Bglap, and Spp1 compared with the cells in basal medium (*p* < 0.05 for all) (Figure 4(a)). Additionally, most of the cells cultured in osteogenic induced medium are positive for Runx2 protein expression (Figure 4(b)). After 21 days of osteogenic induction, Alizarin Red assay showed that osteogenic induced cultures had calcium nodules, while the calcium nodules were absent in the basal cultures (Figure 4(c)).

### 3.5. Adipogenic Differentiation Potential

**Figure 5:**
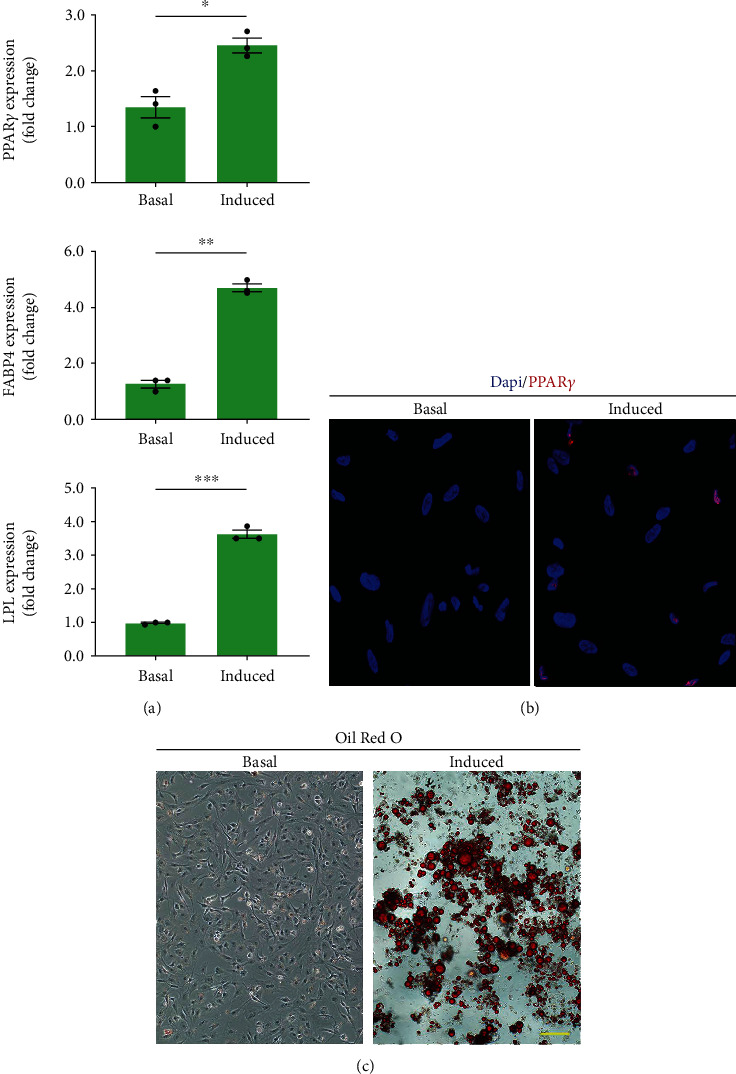
Adipogenic differentiation of urine-derived cells in vitro. (a) adipogenic gene (PPAR*γ*, FABP4, and LPL) expression compared between adipogenic medium and its respective basal cultures. ^∗^*p* < 0.05, ^∗∗∗^*p* < 0.001. PPAR*γ*: peroxisome proliferator-activated receptor *γ*; FABP4: fatty acid-binding protein 4; LPL, lipoprotein lipase. (b) The PPAR*γ* protein expression of the urine-derived cells after 7 days of adipogenic induction. Scale bars = 15 *μ*m. (c) Oil Red O staining of cells after 21 days in adipogenic or basal medium. Intracytoplasmic lipid droplets were seen in the adipogenic induced medium. Scale bars = 20 *μ*m.

After 7 days of adipogenic induced medium, the mRNA expression level of PPAR*γ*, FABP4, and LPL were significantly upregulated in the cells under adipogenic induced medium when compared with the cells under basal medium. Qualitatively, there was a 2.45 ± 0.23, 4.79 ± 0.24 and 3.62 ± 0.21-fold increase for PPAR*γ*, FABP4, and LPL upon adipogenic induction compared to basal cultures, respectively (*p* < 0.05 for all) (Figure 5(a)). Immunofluorescence assay showed that the cells in the adipogenic group expressed the PPAR*γ* obviously, while limited expression was showed in the cells under basal complete medium (Figure 5(b)). Under the influence of adipogenic induction, the isolated cells achieved an adipocytic phenotype by the end of the third week. The presence of intracytoplasmic lipid droplets was confirmed by Oil Red O staining only in induced cultures (Figure 5(c)), but not in basal cultures.

### 3.6. Chondrogenic Differentiation Potential

**Figure 6:**
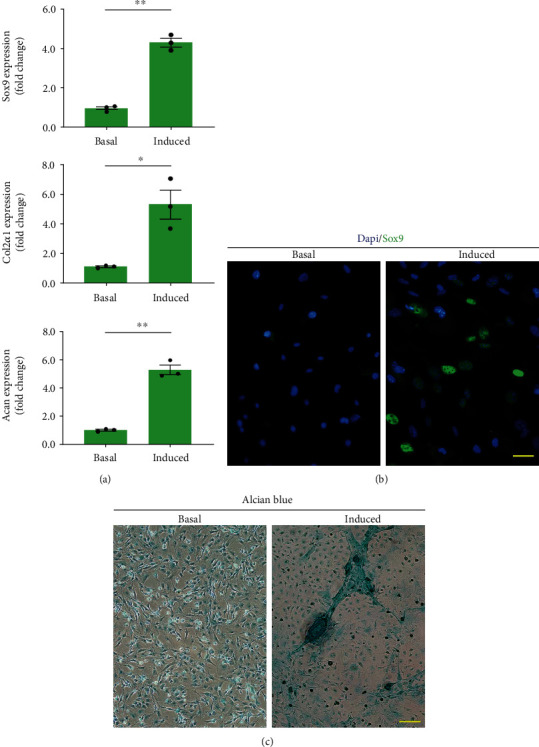
Chondrogenic differentiation of urine-derived cells in vitro. (a) Chondrogenic gene (Sox9, Col2a1, Acan) expression compared between the chondrogenic medium and its respective basal cultures. ^∗^*p* < 0.05, ^∗∗^*p* < 0.01. Sox9, sex-determining region Y-box 9; Col2a1, collagen type II; Acan, Aggrecan. (b) The Sox9 protein expression of the urine-derived cells after 7 days of chondrogenic induction. Scale bars = 15 *μ*m. (c) Alcian blue staining of cells after 21 days in chondrogenic or basal medium. Glycosaminoglycan deposition around the cells was seen in the chondrogenic induced medium. Scale bars = 20 *μ*m.

After 7 days of chondrogenic induction, the increased expression of chondrogenic-associated markers (Sox9, Col2a1, Acan) was found in the chondrogenic group with respect to the control group (*p* < 0.05 for all) (Figure 6(a)). Moreover, immunofluorescence assay showed that the cells cultured in the chondrogenic differentiation medium were positive for Sox9 expression, while no Sox9 expression was found in the cell cultured on basal complete medium (Figure 6(b)). After 21 days of chondrogenic induction, there was glycosaminoglycan deposition found around the cells by Alcian blue staining (Figure 6(c)).

### 3.7. Tenogenic Differentiation Potential

**Figure 7:**
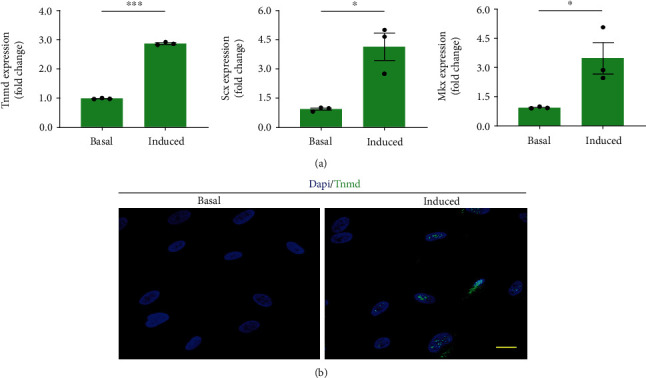
Tenogenic differentiation of urine-derived cells in vitro. (a) Tenogenic gene (Tnmd, Scx, and Mkx) expression compared between tenogenic medium and its respective basal cultures. ^∗^*p* < 0.05, ^∗∗^*p* < 0.01, ^∗∗∗^*p* < 0.001. Tnmd, tenomodulin; Scx, scleraxis; Mkx, mohawk. (b) The Tnmd protein expression of the urine-derived cells after 7 days of tenogenic induction. Scale bars = 15 *μ*m.

The isolated cells exposed to the tenogenic induced medium for 7 days showed a significant enhancement in the Tnmd, Scx, and Mkx expression with respect to the cells under basal cultures (*p* < 0.05 for all) (Figure 7(a)). Immunofluorescence assay showed that the cells in the tenogenic induced medium obviously expressed the Tnmd protein, while limited expression was showed in the cells under basal medium (Figure 7(b)).

## 4. Discussion

MSCs have been isolated from several canine sources, including the bone marrow, adipose tissue, synovium tissue, infrapatellar fat pad, umbilical cord vein, and ovarian tissue [8, 35–38]. In this study, we describe for the first time the isolation, characterization, and differentiation potential of MSCs obtained from canine urine samples, namely, cUSCs. The isolated cUSCs displayed spindle-shaped cells with rapid proliferation potential and were able to self-renewal forming colonies from single cells and showed osteogenic, adipogenic, chondrogenic, and tenogenic differentiation. The protocol developed for isolating cUSCs will be convenient for extrapolating USCs-based therapies from a preclinical canine model for clinical management of tissue injuries.

Plastic adherence is one of the most obvious characteristics of MSCs. Our study showed that the isolated cUSCs adhered to plastic and displayed a spindle-shaped morphology, similar to those reported from humans[15, 39]. Previous studies reported that USCs derived from human urine (hUSCs) proliferated to 70-80% confluence no more than 3 days [16, 40], while the other two studies indicated that USCs derived from humans proliferated to 70-80% confluence no more than 5 days [41, 42]. Similarly, our study showed that after 5 days, the cUSCs proliferated to 70-80% confluence.

Surface markers are another index to identify MSCs. A relative consensus currently exists regarding the surface markers detected with flow cytometry for human MSCs [34]. Unfortunately, a consensus regarding an acceptable flow cytometry profile remains to be determined for canine MSCs. Our study demonstrated that the isolated cell from canine urine was positive for CD29, CD44, CD90, and CD73, while lacking expression of CD45, CD11b, and CD34, which corresponds to the MSC surface markers specified by the International Society for Cellular Therapy (ISCT) [34]. The expressions of these surface markers were consistent with USCs from humans [39, 42].

During osteogenic differentiation of the isolated cUSCs, calcium phosphate deposition and osteogenic-specific gene expressions were tested. Runx2 and Spp1 are known to be upregulated during the osteogenic differentiation of human MSCs [43, 44]. Bglap is a bone-specific gene required for matrix mineralization [45]. In cUSCs with osteogenic induction, Runx2, Spp1, and Bglap were found to be significantly upregulated expressed at day 21, and lots of calcium nodules positive for Alizarin Red staining formed at the culture plate. These results were consistent with similar studies in hUSCs [39].

During the process of cUSC adipogenic differentiation, lipid droplet formation and adipocyte-specific gene (PPAR*γ*, FABP4, and LPL) expression were detected. The cellular function of FABP4 is the coupling of fatty acids to several molecular targets as a fatty acids chaperone [46]. Via peroxisome proliferator response elements, the transcription of FABP4 is directly coupled to PPAR*γ* for enhancing the adipogenic differentiation of MSCs [47]. During the process of adipogenesis, the expression of FABP4 was found to be upregulated in cUSCs. In addition, PPAR*γ* might play a critical role in adipogenesis by binding to the enhancer of adipocyte-specific genes, such as LPL, leptin, fatty acid-binding protein, and the adipocyte P2 gene (aP2) [48, 49]. Thus, the cUSCs under adipogenic induction presented significantly higher expression of PPAR*γ* and LPL with respect to the untreated cUSCs.

As for the chondrogenic differentiation capacity of cUSCs, there exists a dispute. Pei et al. reported that USCs did not present the ability to differentiate into chondrocytes in a 5% O_2_ and 5% CO_2_ incubator up to 14 days [50], while some studies have determined that USCs could differentiate toward the chondrogenic lineages after chondrogenic induction for 28 days [51–53]. Meanwhile, some studies showed that USCs could differentiate into chondrocytes but presented relatively lower chondrogenic potential with respect to MSCs derived from adipose tissue (ASCs) or bone marrow (BMSCs) [40, 54]. But another study indicated that USCs possessed a similar biological characteristic to ASCs and had multilineage differentiation ability [51]. Herein, the chondrogenic potential of cUSCs was showed by a synthesis of proteoglycans using Alcian blue staining. The expressions of Col2a1 and Acan (major cartilage extracellular matrix components) were upregulated in the cUSCs after chondrogenic induction in a 20% O_2_ and 5% CO_2_ incubator. This study indicated that the isolated cUSCs under chondrogenic induction could differentiate into chondrocytes.

BMP-12 is a critical growth factor involved in guiding MSC tenogenic differentiation [55, 56]. Thus, we added it into the culture medium of cUSCs and then determined the tenogenic differentiation potential of cUSCs by evaluating their tenogenic gene (Tnmd, Scx, and Mkx) expression and Tnmd protein expression. Stimulated by BMP-12 for 7 days, the expression level of Tnmd and Scx was significantly upregulated in cUSCs. Scx is critically involved in the development of tendon progenitors, and Mkx is a critical transcription factor for the subsequent tendon differentiation and maturation [57], while the gene expression of Tnmd, a well-known late-stage tenogenic marker [58], was also significantly overexpressed. The enhanced expression of tendon-related genes following BMP-12 treatment is in good agreement with other studies performed on MSCs from humans and rats [55, 56].

A limitation of this study is that the multilineage differentiation potentials of cUSCs were not evaluated in vivo. Previous literatures indicated that the hUSCs have the ability to differentiate into the bone, cartilage, and urinary tract tissue [16, 39, 41]. In future studies, we used the cUSCs combined with tissue-engineering scaffold for regenerating the bone, cartilage, tendon, and urinary tract tissue in the preclinical canine model.

In conclusion, for the first time, cUSC was successfully isolated from canine urine, which presented clonogenicity and high proliferation capacity. In addition, these cells express the specific-makers of MSCs and can differentiate into osteogenesis, chondrogenesis, adipogenesis, and tenogenesis under a specific induction. The successful isolation of USCs from canine urine may help us preliminarily evaluate the efficacy of USCs-based therapy in a preclinical canine model for clinical management of tissue injuries.

## Figures and Tables

**Table 1:** Reagents and formula of the primary medium for canine USCs.

Reagents	Formula (mL)
REGM SingleQuot kit (Lonza, USA)	2.6 mL
DMEM/F-12 (Gibco, USA)	86.4 mL
Fetal bovine serum (Gibco, USA)	10.0 mL
Antibiotic-antimycotic (Gibco, USA)	1.0 mL

**Table 2:** Reagents and formula of proliferation medium for canine USCs.

Reagents	Formula (mL)
DMEM/F-12 (Gibco, USA)	43.5 mL
RE basal medium (Lonza, USA)	42.9 mL
REGM SingleQuot kit (Lonza, USA)	600.0 *μ*L
Fetal bovine serum (Gibco, USA)	10.0 mL
Antibiotic-antimycotic (Gibco, USA)	1.0 mL
GlutaMAX (Gibco, USA)	1.0 mL
MEM non-essential amino acids (Gibco, USA)	1.0 mL
bFGF (Peprotech, USA)	500 ng
PDGF-BB (Peprotech, USA)	500 ng
EGF (Peprotech, USA)	500 ng

**Table 3:** Primer sequences used for qRT-PCR analysis.

Markers	Gene	Primer sequence (5′-3′)
Osteogenic genes	Runx2	Forward CAGACCAGCAGCACTCCATA
		Reverse CAGCGTCAACACCATCATTC
	Bglap	Forward CTGAATCCCGCAAAGGTGGT
		Reverse CTCGTCACAGTTGGGGTTGA
	Spp1	Forward TAGCCAGGACTCCGTTGACT
		Reverse ACACTATCACCTCGGCCATC

Chondrogenic genes	Sox9	Forward GCTCGCAGTACGACTACACTGAC
		Reverse GTTCATGTAGGTGAAGGTGGAG
	Col2a1	Forward GAAACTCTGCCACCCTGAATG
		Reverse GCTCCACCAGTTCTTCTTGG
	Acan	Forward ATCAACAGTGCTTACCAAGACA
		Reverse ATAACCTCACAGCGATAGATCC

Adipogenic genes	PPAR*γ*	Forward primer TCACAGAGTACGCCAAAAGT
		Reverse primer ACTCCCTTGTCATGAATCCT
	FABP4	Forward ATCAGTGTAAACGGGGATGTG
		Reverse GACTTTTCTGTCATCCGCAGTA
	LPL	Forward ACACATTCACAAGAGGGTCAC
		Reverse CTCTGCAATCACACGGATG

Tenogenic genes	Tnmd	Forward GATCCCATGCTGGATGAG
		Reverse TACAAGGCATGATGACACG
	Scx	Forward AAGCTCTCCAAGATCCGAGACACTG
		Reverse AAGAAGGGCCCAGAGTGGC
	Mkx	Forward AGACATGTCATGGCCACAAA
		Reverse TGATGATGAGGGAGACACCA

Housekeeping	GAPDH	Forward CCATCTTCCAGGAGCGAGAT
		Reverse TTCTCCATGGTGGTGAAGAC

## Data Availability

The data used to support the findings of this study are included in the article.

## References

[1] Pantelic M. N., Larkin L. M. (2018). Stem cells for skeletal muscle tissue engineering. *Tissue Engineering. Part B, Reviews*.

[2] Langhans M. T., Yu S., Tuan R. S. (2016). Stem cells in skeletal tissue engineering: technologies and models. *Current Stem Cell Research & Therapy*.

[3] Brown C., McKee C., Bakshi S. (2019). Mesenchymal stem cells: cell therapy and regeneration potential. *Journal of Tissue Engineering and Regenerative Medicine*.

[4] Haynesworth S. E., Goshima J., Goldberg V. M., Caplan A. I. (1992). Characterization of cells with osteogenic potential from human marrow. *Bone*.

[5] YOO J. U. N. G. U., BARTHEL T. R. A. C. I. S., NISHIMURA K. E. I. T. A. (1998). The chondrogenic potential of human bone-marrow-derived mesenchymal progenitor cells. *The Journal of Bone and Joint Surgery. American Volume*.

[6] Wang Q. W., Chen Z. L., Piao Y. J. (2005). Mesenchymal stem cells differentiate into tenocytes by bone morphogenetic protein (BMP) 12 gene transfer. *Journal of Bioscience and Bioengineering*.

[7] Choi Y. H., Kurtz A., Stamm C. (2011). Mesenchymal stem cells for cardiac cell therapy. *Human Gene Therapy*.

[8] Bearden R. N., Huggins S. S., Cummings K. J., Smith R., Gregory C. A., Saunders W. B. (2017). In-vitro characterization of canine multipotent stromal cells isolated from synovium, bone marrow, and adipose tissue: a donor-matched comparative study. *Stem Cell Research & Therapy*.

[9] Wang X., Wang Y., Gou W., Lu Q., Peng J., Lu S. (2013). Role of mesenchymal stem cells in bone regeneration and fracture repair: a review. *International Orthopaedics*.

[10] Pak J., Lee J. H., Kartolo W. A., Lee S. H. (2016). Cartilage regeneration in human with adipose tissue-derived stem cells: current status in clinical implications. *BioMed Research International*.

[11] Qasim M., Chae D. S., Lee N. Y. (2019). Bioengineering strategies for bone and cartilage tissue regeneration using growth factors and stem cells. *Journal of Biomedical Materials Research. Part A*.

[12] Hao Z. C., Wang S. Z., Zhang X. J., Lu J. (2016). Stem cell therapy: a promising biological strategy for tendon-bone healing after anterior cruciate ligament reconstruction. *Cell Proliferation*.

[13] Neo P. Y., Teh T. K. H., Tay A. S. R. (2016). Stem cell-derived cell-sheets for connective tissue engineering. *Connective Tissue Research*.

[14] Xie S., Zhou Y., Tang Y. (2019). Book-shaped decellularized tendon matrix scaffold combined with bone marrow mesenchymal stem cells-sheets for repair of achilles tendon defect in rabbit. *Journal of Orthopaedic Research*.

[15] Ji X., Wang M., Chen F., Zhou J. (2017). Urine-derived stem cells: the present and the future. *Stem Cells International*.

[16] Chen L., Li L., Xing F. (2018). Human urine-derived stem cells: potential for cell-based therapy of cartilage defects. *Stem Cells International*.

[17] Hatsushika D., Muneta T., Nakamura T. (2014). Repetitive allogeneic intraarticular injections of synovial mesenchymal stem cells promote meniscus regeneration in a porcine massive meniscus defect model. *Osteoarthritis and Cartilage*.

[18] BRUDER S. C. O. T. T. P., KRAUS K. A. R. L. H., GOLDBERG V. I. C. T. O. R. M., KADIYALA S. U. D. H. A. (1998). The effect of implants loaded with autologous mesenchymal stem cells on the healing of canine segmental bone defects. *The Journal of Bone and Joint Surgery. American Volume*.

[19] Horie M., Driscoll M. D., Sampson H. W. (2012). Implantation of allogenic synovial stem cells promotes meniscal regeneration in a rabbit meniscal defect model. *The Journal of Bone and Joint Surgery. American Volume*.

[20] Xia X., Chan K. F., Wong G. T. Y. (2019). Mesenchymal stem cells promote healing of nonsteroidal anti-inflammatory drug-related peptic ulcer through paracrine actions in pigs. *Science translational medicine*.

[21] Canapp S. O., Canapp D. A., Ibrahim V., Carr B. J., Cox C., Barrett J. G. (2016). The use of adipose-derived progenitor cells and platelet-rich plasma combination for the treatment of supraspinatus tendinopathy in 55 dogs: a retrospective study. *Frontiers in Veterinary Science*.

[22] Storb R., Epstein R. B., Graham T. C., Thomas E. D. (1970). Methotrexate regimens for control of graft-versus-host disease in dogs with allogeneic marrow grafts. *Transplantation*.

[23] Prentice H. G., Blacklock H. A., Janossy G. (1984). Depletion of T lymphocytes in donor marrow prevents significant graft-versus-host disease in matched allogeneic leukaemic marrow transplant recipients. *Lancet*.

[24] Socie G., Blazar B. R. (2009). Acute graft-versus-host disease: from the bench to the bedside. *Blood*.

[25] Kiviranta I., Tammi M., Jurvelin J., Säämänen A. M., Helminen H. J. (1988). Moderate running exercise augments glycosaminoglycans and thickness of articular cartilage in the knee joint of young beagle dogs. *Journal of Orthopaedic Research*.

[26] Bockstahler B. A., Skalicky M., Peham C., Müller M., Lorinson D. (2007). Reliability of ground reaction forces measured on a treadmill system in healthy dogs. *Veterinary Journal*.

[27] Schreiner A. J., Stannard J. P., Cook C. R. (2020). Comparison of meniscal allograft transplantation techniques using a preclinical canine model. *Journal of Orthopaedic Research*.

[28] Wits M. I., Tobin G. C., Silveira M. D. (2020). Combining canine mesenchymal stromal cells and hyaluronic acid for cartilage repair. *Genetics and Molecular Biology*.

[29] Adams J. E., Zobitz M. E., Reach J. S., An K.-N., Steinmann S. P. (2006). Rotator cuff repair using an acellular dermal matrix graft: an in vivo study in a canine model. *Arthroscopy: The Journal of Arthroscopic & Related Surgery*.

[30] Cook J. L., Smith P. A., Stannard J. P. (2015). A canine hybrid double-bundle model for study of arthroscopic ACL reconstruction. *Journal of Orthopaedic Research*.

[31] Volpon J. B. (1994). Nonunion using a canine model. *Archives of Orthopaedic and Trauma Surgery*.

[32] Harding J., Roberts R. M., Mirochnitchenko O. (2013). Large animal models for stem cell therapy. *Stem Cell Research & Therapy*.

[33] Hoffman A. M., Dow S. W. (2016). Concise review: stem cell trials using companion animal disease models. *Stem Cells*.

[34] Dominici M., Le Blanc K., Mueller I. (2006). Minimal criteria for defining multipotent mesenchymal stromal cells. The International Society for Cellular Therapy position statement. *Cytotherapy*.

[35] Russell K. A., Chow N. H. C., Dukoff D. (2016). Characterization and Immunomodulatory effects of canine adipose tissue- and bone marrow-derived mesenchymal stromal cells. *PLoS One*.

[36] Sasaki A., Mizuno M., Ozeki N. (2018). Canine mesenchymal stem cells from synovium have a higher chondrogenic potential than those from infrapatellar fat pad, adipose tissue, and bone marrow. *PLoS One*.

[37] Hill A. B. T., Therrien J., Garcia J. M., Smith L. C. (2017). Mesenchymal-like stem cells in canine ovary show high differentiation potential. *Cell Proliferation*.

[38] Zucconi E., Vieira N. M., Bueno D. F. (2010). Mesenchymal stem cells derived from canine umbilical cord vein--a novel source for cell therapy studies. *Stem Cells and Development*.

[39] Bento G., Shafigullina A. K., Rizvanov A. A., Sardão V. A., Macedo M. P., Oliveira P. J. (2020). Urine-derived stem cells: applications in regenerative and predictive medicine. *Cells*.

[40] Wu C., Chen L., Huang Y.-z. (2018). Comparison of the proliferation and differentiation potential of human urine-, placenta decidua basalis-, and bone marrow-derived stem cells. *Stem Cells International*.

[41] Guan J., Zhang J., Li H. (2015). Human urine derived stem cells in combination with *β*-TCP can be applied for bone regeneration. *PLoS One*.

[42] Chen A. J., Pi J. K., Hu J. G. (2020). Identification and characterization of two morphologically distinct stem cell subpopulations from human urine samples. *Science China. Life Sciences*.

[43] Liu J., Wu M., Feng G., Li R., Wang Y., Jiao J. (2020). Downregulation of LINC00707 promotes osteogenic differentiation of human bone marrow-derived mesenchymal stem cells by regulating DKK1 via targeting miR‑103a‑3p. *International Journal of Molecular Medicine*.

[44] Nie F., Zhang W., Cui Q., Fu Y., Li H., Zhang J. (2020). Kaempferol promotes proliferation and osteogenic differentiation of periodontal ligament stem cells via Wnt/*β*-catenin signaling pathway. *Life Sciences*.

[45] Carmona H., Valadez H., Yun Y., Sankar J., Estala L., Gomez F. A. (2015). Development of microfluidic-based assays to estimate the binding between osteocalcin (BGLAP) and fluorescent antibodies. *Talanta*.

[46] Furuhashi M., Hotamisligil G. S. (2008). Fatty acid-binding proteins: role in metabolic diseases and potential as drug targets. *Nature Reviews. Drug Discovery*.

[47] Tontonoz P., Spiegelman B. M. (2008). Fat and beyond: the diverse biology of PPAR*γ*. *Annual Review of Biochemistry*.

[48] Rosen E. D., Spiegelman B. M. (2001). PPARgamma: a nuclear regulator of metabolism, differentiation, and cell growth. *The Journal of Biological Chemistry*.

[49] Tontonoz P., Hu E., Graves R. A., Budavari A. I., Spiegelman B. M. (1994). mPPAR gamma 2: tissue-specific regulator of an adipocyte enhancer. *Genes & Development*.

[50] Pei M., Li J., Zhang Y., Liu G., Wei L., Zhang Y. (2014). Expansion on a matrix deposited by nonchondrogenic urine stem cells strengthens the chondrogenic capacity of repeated-passage bone marrow stromal cells. *Cell and Tissue Research*.

[51] Guan J. J., Niu X., Gong F. X. (2014). Biological characteristics of human-urine-derived stem cells: potential for cell-based therapy in neurology. *Tissue Engineering. Part A*.

[52] Bharadwaj S., Liu G., Shi Y. (2013). Multipotential differentiation of human urine-derived stem cells: potential for therapeutic applications in urology. *Stem Cells*.

[53] Gao P., Han P., Jiang D., Yang S., Cui Q., Li Z. (2017). Effects of the donor age on proliferation, senescence and osteogenic capacity of human urine-derived stem cells. *Cytotechnology*.

[54] Kang H. S., Choi S. H., Kim B. S. (2015). Advanced properties of urine derived stem cells compared to adipose tissue derived stem cells in terms of cell proliferation, immune modulation and multi differentiation. *Journal of Korean Medical Science*.

[55] Zarychta-Wiśniewska W., Burdzinska A., Kulesza A. (2017). Bmp-12 activates tenogenic pathway in human adipose stem cells and affects their immunomodulatory and secretory properties. *BMC Cell Biology*.

[56] Dai L., Hu X., Zhang X. (2015). Different tenogenic differentiation capacities of different mesenchymal stem cells in the presence of BMP-12. *Journal of Translational Medicine*.

[57] Liu H., Zhu S., Zhang C. (2014). Crucial transcription factors in tendon development and differentiation: their potential for tendon regeneration. *Cell and Tissue Research*.

[58] Shukunami C., Takimoto A., Oro M., Hiraki Y. (2006). Scleraxis positively regulates the expression of tenomodulin, a differentiation marker of tenocytes. *Developmental Biology*.

